# The Role of IL-33 in Rheumatic Diseases

**DOI:** 10.1155/2013/924363

**Published:** 2013-09-15

**Authors:** Lihua Duan, Jie Chen, Feili Gong, Guixiu Shi

**Affiliations:** ^1^Department of Rheumatology and Clinical Immunology, The First Hospital of Xiamen University, Xiamen 361003, China; ^2^Basic Medical Department of Medical College, Xiamen University, Xiamen 361003, China; ^3^Department of Immunology, Tongji Medical College, Huazhong University of Science and Technology, Wuhan 430030, China

## Abstract

Interleukin-33 (IL-33), a novel member of IL-1 family, has been recently implicated in several inflammatory and autoimmune diseases. IL-33 can be produced by various types of tissues and cells and induce gene expression of Th2-associated cytokines via binding to the orphan receptor ST2. By promoting Th2 type immune response, IL-33 plays important roles in the allergy, whereas its function in autoimmune diseases attracts more attention. Recent studies reported the correlation of IL-33 with rheumatic diseases, and most of them found that the IL-33 expression levels were consistent with disease activity and development. Furthermore, evidence has indicated that IL-33-related treatment may ameliorate the pathogenic conditions and attenuate disease progression of those rheumatic diseases. Therefore, elucidation of the roles of IL-33 in rheumatic diseases would be beneficial to understand the pathogenesis and therapy of these diseases. In this paper, we will summarize the roles of IL-33 in the rheumatic diseases.

## 1. Introduction

IL-33 is a newly reported cytokine of IL-1 family, which has been demonstrated to inducing cytokine syntheses and mediating inflammatory responses through its receptor ST2 [1]. IL-33 is widely expressed in many tissues such as the liver, lung, central nervous system, and multiple types of cells including epithelial cells, endothelial cells, smooth muscle cells, macrophages, and fibroblasts [1–4]. Moreover, IL-33 mainly localizes to the nucleus, but under appropriate signal stimulation such as inflammation, IL-33 is in response processed and passively released from necrotic cells or actively secreted into the extracellular milieu [5] and functions through binding to its receptor ST2 as a proinflammatory cytokine that participates in the development and progression of many diseases, including collagen-induced arthritis [6, 7], anaphylactic shock [8], inflammatory bowel disease [9, 10], autoimmune hepatitis, and ischemia reperfusion injury [11–13]. Here, we will review the role of IL-33 in the pathogenesis of several clinical rheumatic diseases, mainly including rheumatoid arthritis, systemic lupus erythematosus, and ankylosing spondylitis. 

## 2. IL-33 and ST2

IL-33, also named NF-HEV, IL-1F11, is a novel member of IL-1 family which was first reported by Schmitz et al. in 2005. At the protein level, IL-33 is broadly expressed in multiple tissues and organs especially enriched in the central nervous system and gastrointestinal tract [1]. It is considered that the initial translation product is the 30-Kd IL-33 precursor, and following activation of caspase-1, the IL-33 precursor is cleaved, released as an 18-Kd active cytokine [14]. Recent studies report that human IL-33 is processed at Asp178 but not Asp110 as previously claimed and is processed into mature bioactive forms independent of caspase-1 [15, 16]. Recent study also found that IL-33 was mainly localized in the nucleus of cells such as human high endothelial venules cells [3], and its nuclear function was chromatin associated [17, 18]. 

 ST2L, specific receptor of IL-33, is mainly expressed on the surface of Th2 cells, mast cells, and NKT cells, but not on Th1 cells. IL-1R accessory protein (IL-1RAcP) is required for IL-33/ST2L signal transduction, and in IL-1RAcP−/− mouse-derived mast cells, IL-33 failed to induce IL-6 production [19, 20]. IL-33 signals through ERK1/2, p38MAPK, and JNKs [1]. TRAF6 is a critical signal transducer in IL-33 signaling pathway to activate NF-*κ*B and induce Th2 type cytokine production [21]. However, in recent years, ST2L has been reported to be principally expressed on the surface of several other types of cells, such as mast cells [22–24], eosinophils [25–27], basophils [28, 29], and NKT cells [11, 30]. Soluble ST2 (sST2) and transmembrane ST2L arise from a dual promoter system to drive differential mRNA expression [31, 32], and sST2 lacks the transmembrane and cytoplastic domains of ST2L [33]. Therefore, sST2 could serve as an antagonistic decoy receptor of IL-33 and was consistently used to antagonize the effect of IL-33 in experimental studies [34]. 

## 3. IL-33 and Rheumatoid Arthritis

Rheumatoid arthritis (RA) is an autoimmune disease characterized by chronic inflammatory response, including synovial proliferation and excessive proinflammatory cytokine production, leading to eventual cartilage and bone destruction. Several proinflammatory cytokines are considered critical in forming the inflammatory process of RA [35, 36], including IL-1, IL-6, IL-8, IL-15, and TNF-alpha. Blockade of TNF-alpha activity has been widely used in ameliorating RA progression [37–40]. Until now, there has been much evidence confirming the involvement of IL-33 in rheumatoid arthritis. In earlier studies, it was reported that administration of sST2 fusion protein dramatically attenuated disease severity which contains reducing cellular infiltration in the joints, synovial hyperplasia, and joint erosion, by inhibiting the release of proinflammatory cytokines comprising IL-6, IL-12, TNF-alpha, and IFN-gamma [41]. After that, the high expression levels of IL-33 in human RA synovium and experimental arthritis were discovered. Moreover, treatment with an ST2 blocking antibody at the onset of disease attenuated the severity of CIA and reduced joint destruction, which totally suggested that locally produced IL-33 may contribute to the pathogenesis of joint inflammation and destruction [7]. Accordingly, the abnormally elevated level of IL-33 in RA patients was correlated with disease activity compared to the moderate or low activity group or healthy volunteers, and for synovial fluid, IL-33 levels were higher than those in sera. These observations revealed that IL-33 was mainly produced in inflamed joints [42]. Recently, Hong et al. also reported that in patients with RA, the serum level of IL-33 and sST2 was significantly higher than that of healthy controls. Accordingly, in the synovial fluid, the level of IL-33 was significantly higher than that of osteoarthritis patients [43]. All these results confirmed the fact that IL-33/ST2 signaling played a vital role in joint inflammation of human RA and experimental CIA model. 

For the ways by which IL-33/ST2 was involved in the RA pathogenesis, most studies was focused on the relationship between IL-33 and TNF-alpha in RA pathogenesis. For RA patients, by administrating etanercept (a TNF-alpha inhibitor), the serum level of IL-33 significantly decreased at 3 and 6 months, and serum IL-33 levels showed a significant correlation with the number of tender joints, C-reactive protein, Disease Activity Core of 28 joints including CRP and the WBC count, and an inverse correlation with the RBC count and hemoglobin level [44]. This was in accordance with previous studies which reported that TNF-alpha could stimulate the production of IL-33 in vitro [45]. Otherwise, a newly reported study confirmed IL-33 as a target of anti-TNF therapy. They also pointed out that in mouse antigen-induced arthritis (AIA) which resembles human RA, IL-33 could induce and mediate neutrophil migration by activating synoviocytes and macrophages, and this induction was dependent on CXCL1, CCL3, TNF-alpha, and IL-1beta [46]. Furthermore, for patients who do not respond well to TNF-alpha inhibitors treatment, levels of IL-33 showed a significant positive correlation with IL-1beta. Therefore, it is concluded that IL-1beta might be inducing RA inflammation through producing proinflammatory IL-33 [47]. 

## 4. IL-33 and Systemic Lupus Erythematosus (SLE)

Systemic lupus erythematosus (SLE) is a multisystematic autoimmune disease characterized by chronic immune activation and multiple immunologic phenotypes, especially hypergammaglobulinemia and a plethora of autoantibodies [48]. Generally, there was evidence supporting the vital role of IL-33/ST2 signaling in the pathogenesis of SLE. For active SLE patients, the serum sST2 levels were significantly higher than those of inactive patients or healthy controls, whereas IL-33 was not comparable between SLE patients and controls [49]. However, another study discovered that, compared with healthy controls, the level of serum IL-33 was significantly increased in patients with SLE. Furthermore, serum sST2 level showed close correlation with SLEDAI, anti-dsDNA antibody, and prednisolone dosage but negatively with C3, and it was sensitive to change in disease activity longitudinally [50]. The discrepancy of these results may be due to different ways and devices of IL-33 detection. Furthermore, IL-33 level of patients with SLE was closely correlated with ESR, CRP, and IgA but showed significantly independent association of IL-33 with thrombocytopenia, erythrocytopenia, and anti-SSB antibody. Those results suggest that IL-33/ST2 signaling plays a role in SLE in the acute phase. 

## 5. IL-33 and Ankylosing Spondylitis (AS)

Ankylosing spondylitis (AS), characterized by inflammation, bone erosion, and syndesmophyte formation, is a typical and the most common form of seronegative spondyloarthritis. Up to now, numerous studies have investigated the mechanism of AS development. However, there were only a few studies reporting the role of IL-33 in AS so far. It was discovered that in AS patients, serum IL-33 levels were elevated [51]; compared with inactive AS patients, the level of serum IL-33 was significantly higher in patients with active AS [52]. Moreover, serum IL-33 levels were positively correlated with IL-13, IL-4, IL-17, and TNF-alpha levels. Furthermore, these studies showed that IL-33 could enhance TNF-*α* and IL-6 production by peripheral blood mononuclear cells (PBMCs). Besides, neutrophil migration induced by IL-33 in AS patients were observed, which may also be an important mechanism explaining the association between the elevated IL-33 concentrations and AS [52]. Consistently, in RA patients, suppression of ST2 expression in neutrophils reduces Synovial inflammation through preventing IL-33-induced neutrophils migration [46]. 

## 6. Other Rheumatic Diseases

Idiopathic infalmmatory myopathies (IIM), which includes dermatomyositis (DM) and polymyositis (PM), is a chronic systemic disease associated with high morbidity and functional disability. From the immunopathological viewpoint, in both, elevated concentrations of proinflammatory interleukins (TNF, IL-1, IL-6) and increased expression of molecules related to costimulation of T lymphocytes have been described [53]. It is reported that serum sST2 levels were significantly higher in DM and PM patients and correlated with markers of disease activity including CRP, CK, and LDH, and the level of serum sST2 decreased after therapy [54]. This indicates that sST2 may play a role in DM and PM. The role of IL-33 in DM and PM has not been reported yet, but considering the abnormal sST2 expression, it can be inferred that IL-33 may be involved in the pathogenesis of DM and PM. 

Behçet's disease is a systemic inflammatory disorder with recurrent episodes of oral ulceration, skin lesions, genital ulceration, and intraocular inflammation (uveitis). The serum level of IL-33 in active BD patients was significantly higher than that of inactive BD patients or healthy controls. Moreover, IL-33 mRNA expression in the skin lesions of patients with active BD was significantly increased compared to that in healthy skin biopsies. Furthermore, a significant relationship was found between the levels of IL-33 and IL-17 and IL-33 and IL-6 in active BD patients [55]. These indicate that elevated IL-33 level in active BD patients was correlated with disease activity. 

GCA is an inflammatory disease of blood vessels most commonly involving large and medium arteries of the head. Studies have demonstrated that both the innate and adaptive immune system contribute to GCA pathogenesis, such as Th1/Th17 [56, 57]. It was found that IL-33 and ST2 expression was significantly elevated in the inflamed arteries of GCA patients, but it was not accompanied by a concomitant increase of Th2 cytokines whereas elevated expression of IFN-*γ*, p-STAT6 and M2 macrophages polarisation were observed. Although IL-33 primarily induces Th2 immune responses, the role of IL-33 in the inflammation of GCA patients may not relied on inducing Th2 cytokines production, maybe inducing Th1 immune response [58].

Systemic sclerosis (SSc) is a disabling and incurable connective tissue disease with an unknown pathogenesis. In SSc, the combination of vascular abnormalities, collagen deposition, and autoimmunity leads to widespread tissue and organ fibrosis [59]. It has been found that, compared to healthy controls, IL-33 expression was significantly increased in SSc patients. Meanwhile, the serum level of IL-33 was correlated with early disease stage and microvascular involvement [60]. Moreover, some other investigators reported the same observations recently [61]. These data prompted us that IL-33 should be involved in the SSc pathogenesis, and the mechanism may be correlated with the role of IL-33 in promoting fibrosis [62]. 

## 7. Conclusion

Taken together, as a novel member of IL-1 family, IL-33 plays an important role in the development and progression of rheumatic diseases. For the autoimmune diseases above, either IL-33 or ST2 expression was altered in the serum of active patients, and this may be correlated with inflammatory cytokines, such as TNF-alpha and IL-1beta. So far, investigations on IL-33 and rheumatic diseases mostly focus on the expression level of IL-33 and disease activity, but the underlying mechanism and related clinical therapy still remain to be studied. Based on the aforementioned studies, we can infer that the clinical application of IL-33/ST2-related therapy in the treatment patients is full of prospects, although further studies are required to improve the details.
